# F41, a Novel Fungal Indole-Diterpenoid, Overcomes Gemcitabine Resistance in Pancreatic Cancer by Inducing ER Stress

**DOI:** 10.3390/ijms262110694

**Published:** 2025-11-03

**Authors:** Zixiang Gao, Li Liu, Yang Xu, Cong Zhao, Xiaojun Zhao, Ting Zhao, Yongsheng Che, Wuli Zhao

**Affiliations:** 1 State Key Laboratory of Respiratory Health and Multimorbidity, Key Laboratory of Antibiotic Bioengineering, Ministry of Health, Laboratory of Oncology, Institute of Medicinal Biotechnology, Chinese Academy of Medical Sciences, Peking Union Medical College, Beijing 100050, China; s2024010038@student.pumc.edu.cn (Z.G.); kimliu0520@163.com (L.L.); 17860396818@163.com (C.Z.); zhaoxjun00@163.com (X.Z.); 2 Institute of Medicinal Biotechnology Chinese Academy of Medical Sciences, Peking Union Medical College, Beijing 100050, China; bioxuyang@hotmail.com (Y.X.); s2023010029@student.pumc.edu.cn (T.Z.)

**Keywords:** pancreatic cancer, fungal secondary metabolite, endoplasmic reticulum stress, apoptosis

## Abstract

Pancreatic cancer is an aggressive malignancy with a poor prognosis. It is characterized by low surgical resection rates, frequent development of chemoresistance, unsatisfactory treatment outcomes, and a high potential for recurrence and metastasis. Compound F41, a naturally occurring indole-diterpenoid secondary metabolite, was isolated from the entomopathogenic fungus Penicillium sp. Its anti-pancreatic cancer activity has not been previously reported. Our study demonstrates that F41 significantly inhibits DNA replication, invasion, and proliferation in pancreatic cancer cells. By elevating intracellular reactive oxygen species (ROS) levels in pancreatic cancer cells, F41 induces endoplasmic reticulum stress, ultimately leading to apoptosis. These findings suggest that F41 could effectively overcome gemcitabine resistance in a clinical setting, indicating its promise as a potential therapeutic agent for pancreatic cancer.

## 1. Introduction

Pancreatic cancer, a highly aggressive malignancy of the digestive system, claims nearly half a million lives annually worldwide and portends a profoundly poor prognosis. It ranks as the sixth leading cause of cancer-related mortality, accounting for approximately 5% of all global cancer deaths [1]. The past 25 years have witnessed a more-than-twofold increase in the global incidence of pancreatic cancer, presenting a mounting public health burden. Well-documented risk factors for the disease comprise smoking, obesity, diabetes, and chronic alcohol use [2]. The disease is characterized by a dismal median survival of just four months and a five-year survival rate of 13%, with projections indicating it will become the second-leading cause of cancer mortality within the next decade. Typically asymptomatic in its early stages, pancreatic cancer is frequently diagnosed at an advanced and inoperable stage [3]. Consequently, while surgical resection remains the cornerstone of curative-intent treatment, only a small subset of patients qualify. Furthermore, cornerstone chemotherapeutic agents like gemcitabine offer constrained therapeutic benefits. These limitations collectively contribute to the persistently high rates of recurrence and metastasis observed with current treatment modalities [4]. This dire clinical outlook underscores the urgent and unmet need for the development of novel, effective pharmacological therapies.

Natural products serve as a cornerstone of oncology therapeutics, prized for their favorable safety profiles and potent pharmacological activities. Among them, fungal secondary metabolites constitute a vast and largely untapped reservoir of therapeutic candidates. These compounds display unparalleled chemical diversity that eclipses the scope of synthetic chemistry and frequently operate through exquisitely structured bioactive molecules that mediate highly specific and novel mechanisms of action. Consequently, they represent an indispensable strategic asset in confronting future disease challenges, with demonstrated efficacy across antimicrobial, antiviral, and antitumor applications [5].

**Figure 1:**
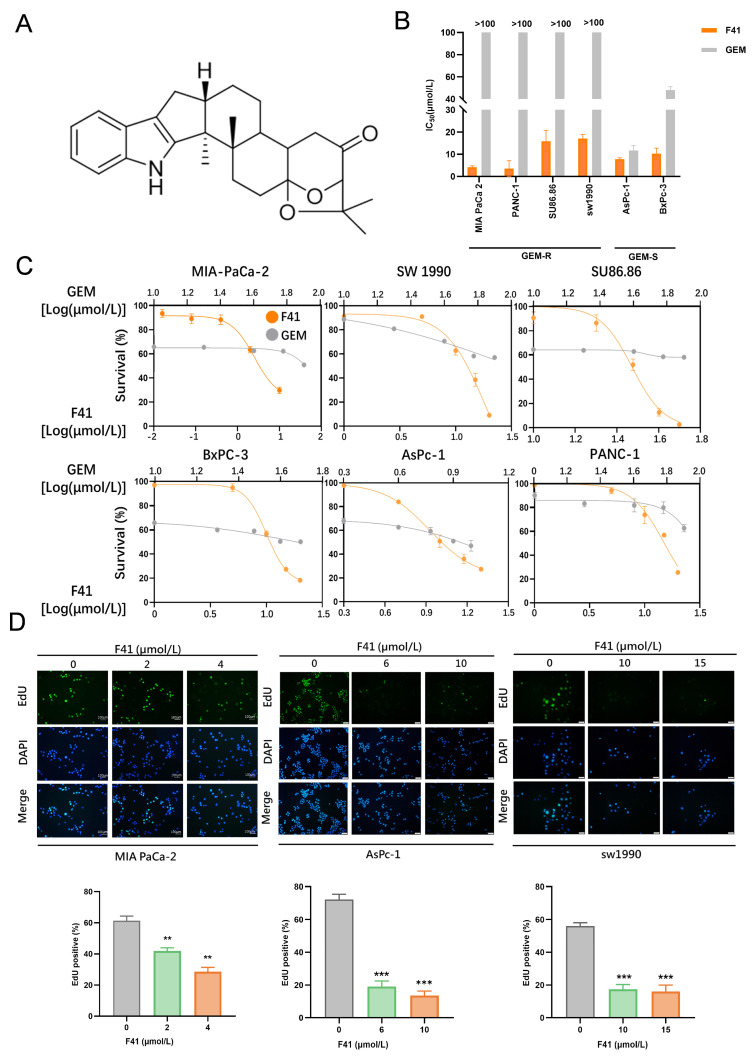
F41 potently suppresses the proliferation of both GEM-resistant and GEM-sensitive pancreatic cancer cells. (**A**) Chemical structure of F41. (**B**) Comparison of IC_50_ values between F41 and gemcitabine (GEM) in various pancreatic cancer cell lines. (**C**) Cell viability measured by MTT assay in different pancreatic cancer cells after 48 h treatment with F41 or gemcitabine. (**D**) EdU colorimetric assay detecting DNA replication in cells treated with varying concentrations of F41 for 24 h. ** *p* < 0.01, and *** *p* < 0.001.

Compound F41 (C_27_H_33_NO_3_) (Figure 1A), an indole-diterpenoid secondary metabolite, was isolated from the entomopathogenic fungus Penicillium sp. found on the cereal cyst nematode [6]. The molecular weight of F41 is 419.56 g/mol. Indole-diterpenoids, exemplified by F41, are a class of secondary metabolites sparsely distributed in fungi, primarily within the genera Claviceps, Aspergillus, Epichloë, and Penicillium [7]. F41 shares the characteristic core scaffold common to all indole-diterpenoids, comprising a cyclic diterpenoid unit fused to an indole moiety. The diterpenoid component is partially derived from the mevalonic acid pathway, where four isoprene units assemble into a twenty-carbon skeleton. This hydrophobic framework dictates the molecule’s fundamental three-dimensional architecture [8]. The indole ring, conversely, originates from the shikimic acid pathway and is derived from tryptophan. This electron-rich planar heterocycle is a critical pharmacophore, frequently engaging in π-π stacking and hydrogen-bonding interactions with biological targets [9]. These two distinct structures are elegantly conjugated via catalysis by fungus-specific enzymes. The parent scaffold is often further decorated with various oxygen-containing functional groups, such as hydroxyl, ketone, and epoxide moieties. These groups not only modulate the molecule’s polarity but, more importantly, serve as key interaction sites for specific binding to biological targets, including ion channels and cellular organelles. Owing to their unique hybrid polycyclic systems and precise stereochemistry, indole-diterpenoids exhibit remarkable and diverse pharmacological activities. For instance, the indole-diterpenoids Paxilline and Nodulisporic Acid are potent modulators of high-conductance calcium-activated potassium (BK) channels and glutamate-gated chloride channels (GluCls), respectively. Their complex three-dimensional structures enable highly specific recognition of these ion channels, underpinning their therapeutic potential for treating neurological disorders such as essential tremor, epilepsy, and anxiety [10]. Other indole-diterpenoids demonstrate additional pharmacologic effects, including antibacterial and antiviral activities, as well as inhibition of protein tyrosine phosphatase activity [11]. Thus, indole-diterpenoids, represented by F41, constitute a highly promising family of natural products with significant medicinal value, positioning them as a crucial source for innovative drug discovery. However, the antitumor activity of F41, particularly against pancreatic cancer, had remained unreported. Therefore, we investigated the efficacy and underlying mechanisms of F41 against pancreatic cancer. Herein, we report for the first time the potent anti-pancreatic cancer activity of this fungus-derived indole-diterpenoid, F41.

## 2. Results

### 2.1. F41 Potently Suppresses the Proliferation of Both GEM-Resistant and GEM-Sensitive Pancreatic Cancer Cells

Although gemcitabine (GEM) is widely used in the clinical treatment of pancreatic cancer, its efficacy is limited by drug resistance. In a screen of fungal metabolites for anticancer activity, we identified compound F41, which exhibited potent antitumor effects in both GEM-resistant (GEM-R) and GEM-sensitive (GEM-S) pancreatic cancer cells. Notably, its anti-pancreatic cancer activity has not been previously reported since its isolation.

We first evaluated the cytotoxic activity of F41 against a panel of six pancreatic cancer cell lines, including four GEM-R lines (MIA PaCa-2, PANC-1, SW 1990, and SU86.86) and two GEM-S lines (BxPC-3 and AsPC-1). The cells were treated with varying concentrations of F41 for 48 h, and cell viability was assessed using the MTT assay. The results demonstrated that F41 significantly inhibited the proliferation of pancreatic cancer cells in a dose-dependent manner, and the half-maximal inhibitory concentration (IC_50_) was calculated from the cell survival curves. In parallel, based on the established literature, we also tested GEM across a range of concentrations to determine its cytotoxic effects and corresponding IC_50_ values in the same GEM-S and GEM-R cell lines [12]. Comparative analysis revealed that F41 exhibited superior anti-proliferative activity compared to gemcitabine (Figure 1B,C). Notably, F41 also showed potent antitumor effects against GEM-R cells, demonstrating its ability to effectively overcome GEM resistance.

To visualize DNA replication, we employed an EdU incorporation assay to evaluate the effect of F41 on DNA synthesis in pancreatic cancer cells. The treatment of MIA PaCa-2, SW 1990, and AsPC-1 cells with increasing concentrations of F41 (2, 4, 6, 10, 15, and 20 μmol/L) resulted in a dose-dependent decrease in the percentage of EdU-positive cells, indicating a strong inhibition of DNA replication (Figure 1D).

In summary, these findings demonstrate that the fungal natural product F41 potently suppresses the proliferation of both GEM-R and GEM-S pancreatic cancer cells and may reverse GEM resistance.

### 2.2. F41 Inhibits Pancreatic Cancer Cell Migration and Clonogenicity

**Figure 2:**
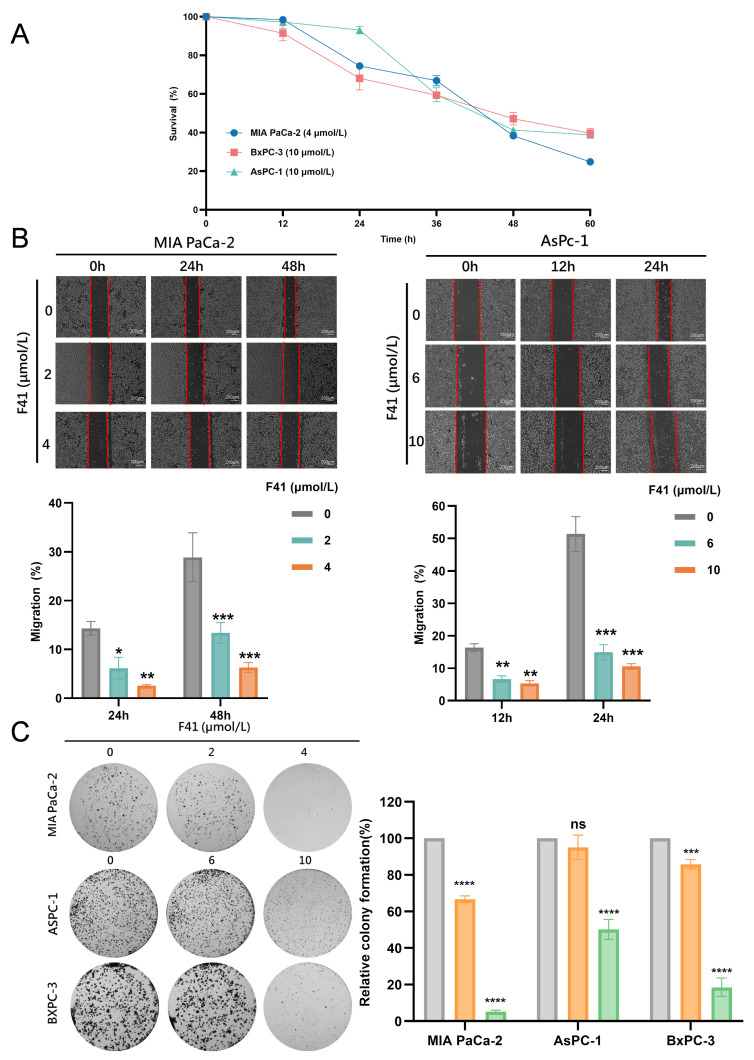
F41 inhibits pancreatic cancer cell migration and clonogenicity. (**A**) Viability of AsPC-1, MIA PaCa-2, and BxPC-3 cells following treatment with F41 for different durations, as determined by MTT assay. (**B**) Migration of cells treated with various concentrations of F41 at different time points. (**C**) Colony formation ability of cells after 7 days of treatment with different concentrations of F41. ns, no significance, * *p* < 0.05, ** *p* < 0.01, *** *p* < 0.001 and **** p < 0.0001.

We next evaluated the time-dependent effects of F41 by treating MIA PaCa-2, BxPC-3, and AsPC-1 cells with F41 (4 and 10 μmol/L) for 12, 24, 36, 48, and 60 h, followed by an assessment of cell viability using the MTT assay. The results revealed a clear time-dependent inhibitory effect of F41 on pancreatic cancer cells (Figure 2A).

To simulate tumor cell migration, we performed wound healing assays. MIA Pa-Ca-2 and AsPC-1 cells were treated with various concentrations of F41 (2, 4, 6, and 10 μmol/L), and images were captured at 0, 12, 24, and 48 h to calculate migration rates. Compared to the control, under low-serum (3% FBS) conditions, F41 significantly inhibited cell migration (Figure 2B).

Furthermore, we employed a clonogenic assay to examine the effect of F41 on the colony-forming ability of pancreatic cancer cells, which reflects their proliferative potential, invasiveness, and sensitivity to cytotoxic agents. Cells (MIA PaCa-2, BxPC-3, and AsPC-1) were treated with different concentrations of F41 (2, 4, 6, and 10 μmol/L) for 7 days, after which the colonies were counted. The results demonstrated that F41 dose-dependently inhibited clonogenic survival (Figure 2C). At concentrations as low as 4 and 6 μmol/L, colony formation was reduced to less than 50% of that in the control group, indicating a potent inhibitory effect of F41 on pancreatic cancer cells.

### 2.3. F41 Induces Apoptosis Through ROS Generation

**Figure 3:**
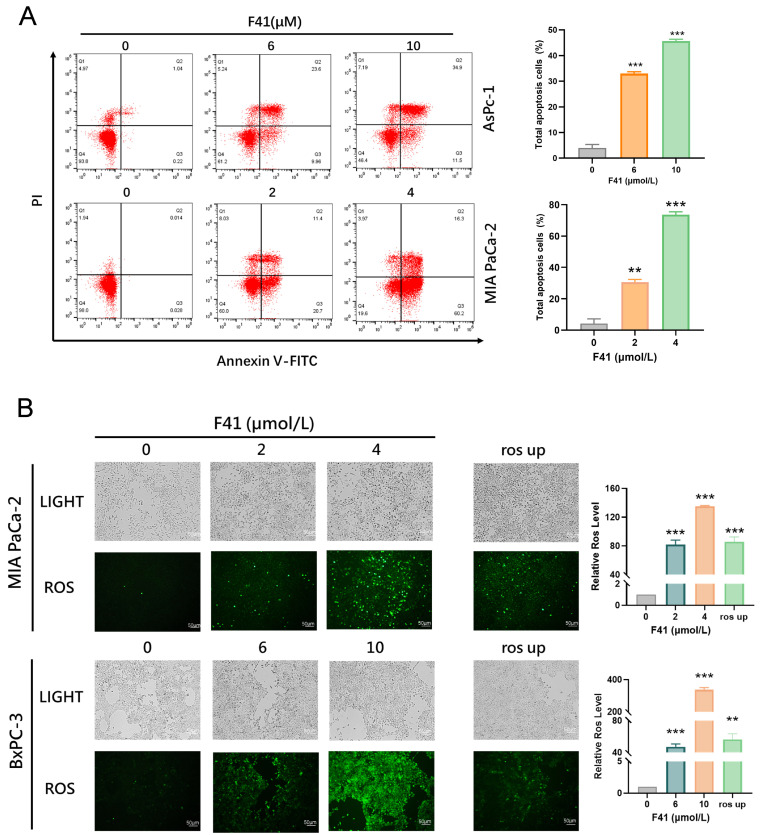
F41 induces apoptosis through reactive oxygen species (ROS) generation. (**A**) Apoptosis detection by flow cytometry in AsPC-1 and MIA PaCa-2 cells treated with F41 and stained with Annexin V-FITC and propidium iodide (PI). (**B**) Intracellular ROS levels detected by DCFH-DA probe and visualized under a fluorescence microscope in BxPC-3 and MIA PaCa-2 cells after F41 treatment. ** *p* < 0.01, and *** *p* < 0.001.

The above results demonstrate that F41 inhibits the DNA replication, migration, and clonogenic ability of tumor cells in dose- and time-dependent manners. To further investigate the antitumor mechanism of F41, we treated MIA PaCa-2 and AsPC-1 cells with different concentrations of F41 (2, 4, 6, and 10 μmol/L) and analyzed apoptosis by flow cytometry. The results showed that F21 significantly induced apoptosis in a concentration-dependent manner (Figure 3A).

Apoptosis, a form of programmed cell death, plays a crucial role in maintaining homeostasis, development, and various pathological conditions. Reactive oxygen species (ROS) are highly reactive molecules, and studies have shown that ROS accumulation triggers oxidative stress, leading to mitochondrial damage and subsequent induction of apoptosis [13]. We therefore measured intracellular ROS levels following F41 treatment and found that they increased significantly with rising concentrations of F41 (Figure 3B).

### 2.4. F41 Induces Endoplasmic Reticulum Stress in Pancreatic Cancer Cells

**Figure 4:**
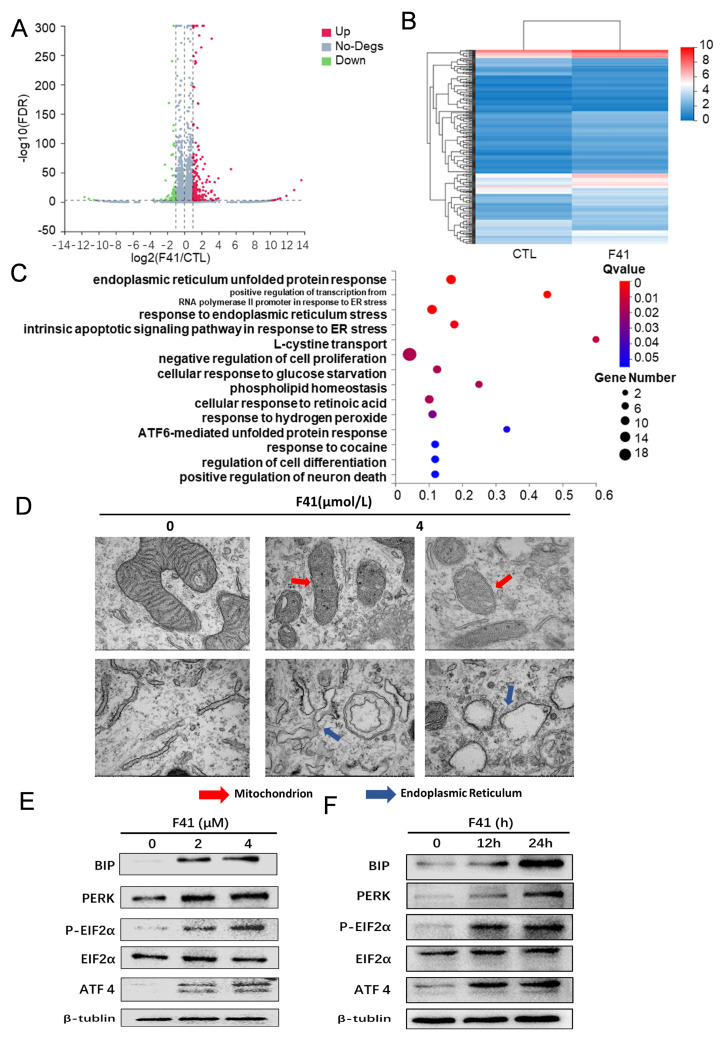
F41 induces endoplasmic reticulum stress in pancreatic cancer cells. (**A**,**B**) Transcriptomic analysis of MIA PaCa-2 cells treated with F41. (**A**) Volcano plot and (**B**) heatmap of differentially expressed genes (DEGs). (**C**) Gene Ontology (GO) enrichment analysis of DEGs, showing significant enrichment in endoplasmic reticulum (ER) stress-related pathways. (**D**) Ultrastructural changes in MIA PaCa-2 cells after F41 treatment observed under transmission electron microscopy. Swollen ER (blue arrows) and damaged mitochondria (red arrows) are indicated. (**E**,**F**) Western blot analysis of ER stress-related protein expression in MIA PaCa-2 cells following F41 treatment.

To further elucidate the mechanism underlying the cytotoxic effects of F41 on pancreatic cancer cells, we performed transcriptome sequencing on both untreated and F41-treated MIA PaCa-2 cells. Using next-generation sequencing technology, we comprehensively profiled the entire transcriptome, including mRNA, ribosomal RNA, and non-coding RNA. The analysis revealed 314 differentially expressed genes (DEGs) in F41-treated cells compared to the untreated controls, comprising 245 up-regulated and 69 down-regulated genes (Figure 4A,B). Subsequent Gene Ontology (GO) enrichment analysis of these DEGs indicated significant enrichment in biological processes such as “endoplasmic reticulum unfolded protein response,” “positive regulation of transcription from RNA polymerase II promoter in response to ER stress,” “response to endoplasmic reticulum stress,” and “intrinsic apoptotic signaling pathway in response to ER stress” (Figure 4C). The endoplasmic reticulum (ER) is a critical organelle involved in protein folding, secretion, and lipid biosynthesis. ER stress is triggered when proteostasis is disrupted due to the accumulation of misfolded or unfolded proteins, and severe ER dysfunction can ultimately lead to apoptosis. These findings led us to hypothesize that F41 induces apoptosis in pancreatic cancer cells by activating ER stress.

To validate this, we examined ultrastructural changes in MIA PaCa-2 cells using transmission electron microscopy (TEM). Compared to the untreated controls, F41-treated cells exhibited significant ER swelling, accompanied by the disruption of mitochondrial cristae and overall mitochondrial integrity—morphological features consistent with ER stress (Figure 4D).

We further investigated the expression of key signaling molecules associated with ER stress. During ER stress, the PERK/EIF2α/ATF4 pathway is activated, along with up-regulation of the ER chaperone protein BIP [14]. PERK is a cytoplasmic protein kinase that phosphorylates eIF2α and enhances ATF4 expression under stress conditions. BIP, one of the most abundant ER proteins, plays a central role in the unfolded protein response. Western blot analysis showed that treatment with increasing concentrations of F41 (2 and 4 μmol/L) or extended exposure times (12 and 24 h at 4 μmol/L) led to dose- and time-dependent up-regulation of BIP and components of the PERK/EIF2α/ATF4 pathway (Figure 4E).

Collectively, these findings from transcriptomic profiling, electron microscopy, and molecular expression analyses demonstrate that F41 induces endoplasmic reticulum stress in pancreatic cancer cells, ultimately leading to apoptosis.

### 2.5. NAC Partially Reverses F41-Induced Proliferation Inhibition, ER Stress, and Apoptosis

**Figure 5:**
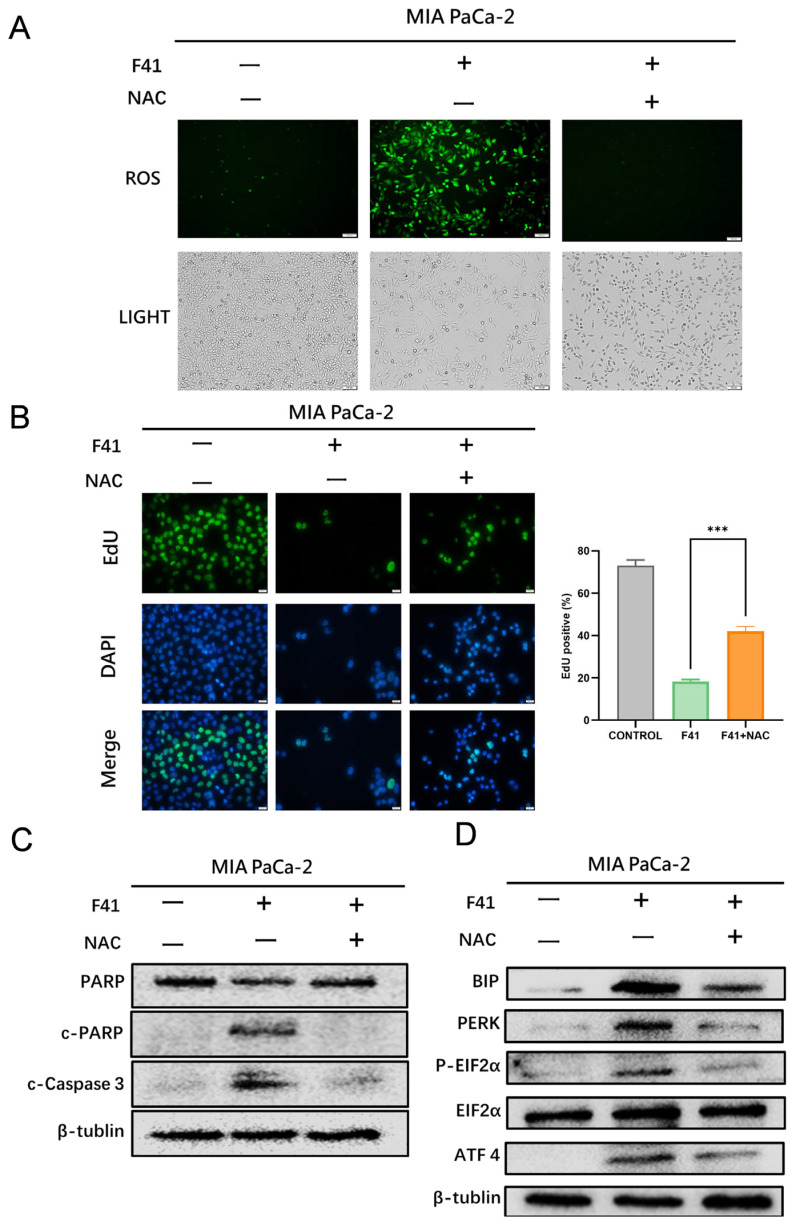
NAC partially reverses F41-induced proliferation inhibition, ER stress, and apoptosis. (**A**) Intracellular ROS levels in MIA PaCa-2 cells after co-treatment with F41 and NAC. (**B**) DNA replication assessed by EdU assay in MIA PaCa-2 cells following co-treatment with F41 and NAC. (**C**) Western blot analysis of apoptosis-related proteins in MIA PaCa-2 cells after co-treatment with F41 and NAC. (**D**) Western blot analysis of endoplasmic reticulum (ER) stress-related proteins in MIA PaCa-2 cells following co-treatment with F41 and NAC. *** *p* < 0.001.

To further validate the role of ROS elevation in F41-induced endoplasmic reticulum (ER) stress and apoptosis, we conducted rescue experiments using N-acetylcysteine (NAC). NAC, a precursor of glutathione, plays a critical role in cellular antioxidant defense. It can be deacetylated to form cysteine, which is subsequently utilized for glutathione synthesis. Additionally, NAC contains a thiol group that directly scavenges ROS, thereby alleviating oxidative stress and cellular damage. To determine whether ROS accumulation contributes to F41-induced ER stress and apoptosis, pancreatic cancer cells were pre-treated with NAC (10 mmol/L) followed by co-treatment with F41. Intracellular ROS levels were significantly reduced in the NAC + F41 group compared to cells treated with F41 alone, confirming the efficacy of NAC in mitigating F41-induced oxidative stress (Figure 5A).

We next evaluated DNA synthesis using EdU incorporation assays and found that NAC pre-treatment partially restored DNA synthesis and reversed the anti-proliferative effect of F41 (Figure 5B). To further investigate the effect of NAC on ER stress and apoptosis, we examined the expression of related proteins by Western blotting. The results demonstrated that NAC attenuated F41-induced activation of the PERK/EIF2α/ATF4 pathway and the expression of the ER chaperone BIP. Furthermore, NAC reduced the levels of apoptosis-related markers, including cleaved PARP and cleaved caspase-3 (Figure 5C).

These findings indicate that NAC can partially reverse F41-induced ER stress and apoptosis. Taken together, our results suggest that F41 induces ER stress and subsequent apoptosis in pancreatic cancer cells primarily through ROS generation, and that these effects can be partially rescued by the antioxidant NAC.

## 3. Discussion

Pancreatic cancer is one of the most aggressive and lethal solid malignancies. Although surgical resection can be effective, its impact on overall survival remains minimal due to the high rates of recurrence and metastasis [15]. While gemcitabine-based monotherapy or combination chemotherapy may improve patient prognosis to some extent [16], the development of drug resistance often hinders long-term treatment efficacy [17]. Thus, there is an urgent need for novel therapeutic agents against pancreatic cancer. Natural products derived from plants and microorganisms have attracted increasing attention for their unique pharmacological activities and their ability to induce tumor cell death through multiple pathways, including endoplasmic reticulum (ER) stress-mediated apoptosis, the extrinsic death receptor pathway, and the intrinsic mitochondrial pathway, demonstrating considerable anticancer potential [18].

Compound F41 is an indole-diterpenoid derived from fungal secondary metabolites—a diverse group of natural products with various structures and significant antitumor activities. For instance, Plinabulin, from the marine fungus Aspergillus sp., targets tubulin and inhibits mitosis, offering a treatment strategy for non-small-cell lung cancer [19]. Versicolactone B, isolated from a marine-derived strain of Aspergillus versicolor, exhibits potent cytotoxicity across multiple cancer cell lines. It induces S-phase arrest and promotes the formation of apoptotic bodies and nuclear condensation in PANC-1 pancreatic cancer cells, ultimately leading to apoptosis [20]. These findings prompted us to investigate the anti-pancreatic cancer effects of F41.

Our results demonstrate that F41 exhibits superior potency over gemcitabine, with significantly lower IC_50_ values against both gemcitabine-resistant (GEM-R; MIA PaCa-2, PANC-1, SW 1990, and SU86.86) and gemcitabine-sensitive (GEM-S; BxPC-3 and AsPC-1) cell lines. This positions F41 as a promising therapeutic candidate capable of potentially overcoming gemcitabine resistance in clinical settings. Furthermore, functional assays—including EdU incorporation, colony formation, and wound healing—consistently revealed that F41 concentration- and time-dependently suppresses DNA replication, clonogenic survival, and migratory capacity in pancreatic cancer cells, irrespective of their gemcitabine sensitivity. 

Having established the efficacy of F41, we next explored its underlying mechanism. Our data reveal that F41 induces apoptosis in pancreatic cancer cells by significantly elevating intracellular ROS levels. This observation is consistent with established knowledge that external stimuli can trigger mitochondrial ROS generation, leading to oxidative stress and subsequent apoptosis, thereby implicating this pathway in the mode of action of F41 [21]. We therefore propose that F41 exerts its antitumor effects by inducing radical oxygen species production and apoptotic cell death. Transcriptome sequencing and transmission electron microscopy revealed that F41 induces ER stress and causes structural damage to both the ER and mitochondria. ER stress has been implicated in various diseases, including cancer, metabolic disorders, and chronic inflammation, and has emerged as a target for small molecules and gene therapies, highlighting its therapeutic relevance [22]. 

Using the ROS scavenger NAC, we demonstrated that NAC attenuates F41-induced proliferation inhibition and reverses ROS accumulation, apoptosis, and ER stress activation. Previous studies have shown that natural compounds such as curcumin and schisandrin A can induce ROS-dependent ER stress, leading to tumor cell apoptosis [23,24]. Collectively, our findings support the conclusion that F41 induces apoptosis in pancreatic cancer cells through ROS-dependent ER stress activation, and that these effects can be partially reversed by NAC.

## 4. Materials and Methods

### 4.1. Cell Culture

The human pancreatic cancer cell lines employed in this research (AsPC-1, BxPC-3, MIA PaCa-2, Panc-1, SW1990, and SU86.86) were acquired from the Institute of Basic Medical Sciences, Chinese Academy of Medical Sciences (Beijing, China). These cells were grown in DMEM or RPMI-1640 medium containing 10% fetal bovine serum (FBS) and 1% penicillin–streptomycin. All cell lines were kept in a humidified incubator at 37 °C with 5% CO_2_ for routine propagation.

### 4.2. Cell Viability Assay (MTT)

Cells in the logarithmic growth phase were plated in 96-well plates (NEST, Wuxi, China) at 7 × 10^3^ cells per well in 200 μL of complete medium for a 24 h incubation at 37 °C with 5% CO_2_. Following this, we replaced the medium with fresh medium containing serially diluted test compounds. After a 48 h treatment period, we carefully removed the medium and added 150 μL of MTT solution (0.5 mg/mL in PBS; Sigma-Aldrich, St. Louis, MO, USA) to each well. The plates were then returned to the incubator for 4 h. Next, the MTT solution was aspirated, and we dissolved the resulting formazan crystals in 150 μL of dimethyl sulfoxide (DMSO). Absorbance was read at 490 nm using a BioTek ELx800 microplate reader (BioTek Instruments, Winooski, VT, USA), with all experiments including a minimum of three biological replicates.

### 4.3. Colony Formation Assay

Cells were plated in 6-well plates at a density of 1000 cells per well and given 24 h to adhere. Following this attachment period, the culture medium was replaced with a drug-containing medium. The cells were then cultured for 7 days. After this incubation, the cells were washed twice with phosphate-buffered saline (PBS; Zhongke Maichen, Beijing, China, ZM-001), fixed with 4% paraformaldehyde (Beyotime, Shanghai, China, P0099) for 15 min at room temperature, and stained using 0.1% crystal violet (Beyotime, Shanghai, China, C0121) for another 15 min. Excess stain was rinsed off with distilled water, and the plates were air-dried prior to being scanned. Colonies were assessed, and those containing more than 50 cells were counted.

### 4.4. Western Blot Analysis

Following a 24 h adherence period after seeding in 6-well plates at 2.5 × 10^5^ cells/well, cells were treated as required. Subsequently, the cells were lysed on ice for 30 min using RIPA buffer (Beyotime, Shanghai, China, P0013B) containing protease and phosphatase inhibitors (Beyotime, Shanghai, China, P1081). The resulting lysates were then centrifuged at 12,000× *g* for 20 min at 4 °C to collect the supernatant. Protein concentration in the supernatants was quantified with a BCA assay kit (Beyotime, Shanghai, China, P0012). Equal protein aliquots (20 μg per lane) were resolved by 10% SDS-PAGE and electrophoretically transferred onto PVDF membranes (Millipore, Burlington, MA, USA, IPVH00010). The membranes were blocked with 5% non-fat milk for 1 h at room temperature before being probed with primary antibodies (diluted 1:1000 in TBST) overnight at 4 °C. After washing, the membranes were incubated with HRP-conjugated secondary antibodies (1:5000) for 2 h at room temperature. Finally, protein bands were detected using an ECL substrate (Millipore, Burlington, MA, USA, WBKLS0100).

### 4.5. Apoptosis Analysis by Flow Cytometry

The assessment of cell apoptosis was conducted using a commercial Annexin V-FITC/PI double-staining kit in accordance with the provided instructions. Post-treatment, cells were harvested, subjected to PBS washing, and resuspended to 1 × 10^6^ cells/mL. A 100 μL sample of the suspension was then co-stained with 5 μL of Annexin V-FITC and 10 μL of propidium iodide (PI), followed by a 20 min incubation in darkness. Flow cytometric analysis was immediately performed after incubation, and the acquired data were analyzed with the FlowJo software (Version 10.8.1, Ashland, OR, USA).

### 4.6. Wound Healing Assay

A cell migration assay was performed using the wound healing method. Briefly, cells were seeded in 6-well plates at 4–5 × 10^5^ cells per well and cultured to ~70% confluence. A linear wound was created in the monolayer with a sterile 200 μL pipette tip. Post-washing with PBS, the cells were cultured in a low-serum medium (3% FBS). Wound closure was monitored by imaging identical locations at 0, 12, 24, and 48 h with an inverted microscope. The acquired images were analyzed with the ImageJ (Version 1.54, Washington, DC, USA) software to calculate the percentage of wound closure.

### 4.7. Cell Proliferation Assay (EdU)

Cell proliferation was assessed with the EdU Apollo488 In Vitro Kit. Cells were plated on coverslips in 24-well plates at a density of 2–4 × 10^4^ cells per well. Following a 24 h drug treatment, the cells were pulsed with 50 μM EdU working solution for 2 h. The cells then underwent a series of processing steps: fixation with 4% paraformaldehyde for 15 min, permeabilization with 0.25% Triton X-100 for 20 min, and finally fluorescent staining involving the Apollo reaction and DAPI nuclear counterstain for 30 min. Fluorescence microscope images were acquired from random fields, and the proportion of EdU-positive cells was quantified.

### 4.8. Transmission Electron Microscopy (TEM)

Ultrastructural alterations in F41-treated cells were investigated by transmission electron microscopy (TEM). MIA PaCa-2 cells were plated in 6-well plates at 2 × 10^5^ cells per well and cultured for 24 h. After attachment, the cells were exposed to fresh medium containing F41, with untreated cells serving as negative controls. Following a 24 h incubation, the cells were harvested by trypsinization after PBS washing and pelleted by centrifugation at 800× *g* for 5 min. The cell pellets underwent primary fixation with 2.5% glutaraldehyde, followed by post-fixation in 1% osmium tetroxide containing 1.5% potassium ferrocyanide for 1 h. Subsequently, the samples were dehydrated through a graded ethanol series (50% to 100%), and en bloc staining was performed with 2% uranyl acetate in 70% ethanol at room temperature. After dehydration and staining, the samples were embedded in epoxy resin. Ultrathin sections (60 nm) were cut using an ultramicrotome, mounted on copper grids, and finally observed under a JEM-1400 Plus transmission electron microscope (JEOL, Tokyo, Japan) at an accelerating voltage of 120 kV.

### 4.9. Transcriptome Sequencing and Analysis

Total RNA was extracted from control and F41-treated cells using a TRIzol reagent (Carlsbad, CA, USA). After quality control, cDNA library construction and sequencing were performed on the BGISEQ-500RS platform by BGI (Shenzhen, China). Raw sequencing data were quality-controlled and aligned to the reference genome. Gene expression levels were quantified using the RSEM software (Version 1.2.12, University of Wisconsin-Madison, WI, USA). Differentially expressed genes (DEGs) were identified using thresholds of FDR < 0.001 and |log2(fold change)| > 1. Gene Ontology (GO) functional enrichment analysis was performed using the Phyper algorithm with Bonferroni correction (Q-value ≤ 0.05 considered significant).

### 4.10. Intracellular ROS Detection

Intracellular ROS production was assessed with the DCFH-DA probe. Briefly, after plating in 6-well plates at a density of 2.5 × 10^5^ cells per well and reaching adherence, cells were subjected to a 24 h drug treatment. The cells were then loaded with 10 μM DCFH-DA by incubation for 20 min at 37 °C in the dark. Following three washes with serum-free medium to remove excess probe, fluorescence was detected and imaged using a fluorescence microscope, or quantified by flow cytometry. In this experiment, Rosup treatment was utilized to establish the positive control.

### 4.11. Reagents and Instruments

DMEM/RPMI-1640 medium and fetal bovine serum were purchased from Gibco (Grand Island, NY, USA). Penicillin–streptomycin, trypsin-EDTA, and PBS were products of Zhongke Maichen Technology (Beijing, China). The TRIzol was obtained from Invitrogen (Carlsbad, CA, USA). The EdU detection kit was obtained from RiboBio (Guangzhou, China). The ROS detection kit, Annexin V-FITC Apoptosis Detection Kit, RIPA lysis buffer, BCA protein assay kit, and others were purchased from Beyotime Biotechnology (Nantong, China). The primary antibodies against PERK (C33E10) and BIP (C50B12) were from Cell Signaling Technology (Danvers, MA, USA). The primary antibodies against EIF2α, p-EIF2α, ATF4, cleaved PARP, PARP, and β-tubulin were from Beyotime (Nantong, China). HRP-conjugated secondary antibodies were from Zhongshan Golden Bridge (Beijing, China). ECL chemiluminescence substrate was from Millipore (Burlington, Massachusetts, USA). MTT, DMSO, N-acetylcysteine (NAC), and other reagents were from Sigma-Aldrich (St. Louis, MO, USA). DAPI staining solution was from Invitrogen (Carlsbad, CA, USA). Major instruments used included an Olympus inverted microscope IX73 (Olympus Corporation, Tokyo, Japan), BD FACSCalibur flow cytometer (Becton, Dickinson and Company, Franklin Lakes, NJ, USA), BioTek ELx800 microplate reader (BioTek Instruments, Winooski, VT, USA), Thermo cell culture incubator (Thermo Fisher Scientific, Waltham, MA, USA), and SpectraMax chemiluminescence imaging system (Molecular Devices, San Jose, CA, USA).

### 4.12. Statistical Analysis

All experiments were independently repeated at least three times. Data are presented as mean ± standard deviation. Comparisons between groups were performed using the unpaired Student’s *t*-test or one-way analysis of variance (ANOVA), with appropriate post hoc tests. A *p*-value of less than 0.05 was considered statistically significant. All statistical analyses were conducted using the GraphPad Prism software (Version 10.0, La Jolla, CA, USA).

## 5. Conclusions

Compound F41, a natural fungal secondary metabolite featuring an indole-diterpenoid structure, exhibits significant anti-pancreatic cancer potential by potently suppressing DNA replication, migration, and proliferation of cancer cells. Its anticancer effects are mediated through the elevation of intracellular ROS levels, induction of endoplasmic reticulum stress, and subsequent activation of apoptosis. These attributes position F41 as a promising candidate for the treatment of pancreatic cancer.

## Data Availability

The data that support the findings of this study are available from the corresponding author upon reasonable request.
